# Bleomycin-enhanced alternative splicing of fibroblast growth factor receptor 2 induces epithelial to mesenchymal transition in lung fibrosis

**DOI:** 10.1042/BSR20180445

**Published:** 2018-11-14

**Authors:** Kui-Jun Chen, Qing Li, Chang-Mei Weng, Zhao-Xia Duan, Dong-Dong Zhang, Zhi-Qiang Chen, Jing Chen, Jian-Min Wang

**Affiliations:** 1 Research Institute of Surgery, Daping Hospital, Third Military Medical University, Chongqing 400042, People’s Republic of China; 2 State Key Laboratory of Trauma, Burn and Combined Injury, Chongqing 400042, People’s Republic of China; 3 Cancer Center, Daping Hospital and Research Institute of Surgery, Third Military Medical University, Chongqing 400042, People’s Republic of China

**Keywords:** alternative splicing, epithelial splicing regulatory proteins, fibroblast growth factor receptor, idiopathic pulmonary fibrosis, TGF-β/Smad signaling pathway

## Abstract

Idiopathic pulmonary fibrosis (IPF) is an important public health problem, and it has few treatment options given its poorly understood etiology; however, epithelial to mesenchymal transition (EMT) of pneumocytes has been implicated as a factor. Herein, we aimed to explore the underlying mechanisms of lung fibrosis mediated by EMT, with a focus on the alternative splicing of fibroblast growth factor receptor 2 (FGFR2), using bleomycin (BLM)-induced lung fibrotic and transgenic mouse models. We employed BLM-induced and surfactant protein C (SPC)-Cre and LacZ double transgenic mouse models. The results showed that EMT occurred during lung fibrosis. BLM inhibited the expression of epithelial splicing regulatory protein 1 (ESRP1), resulting in enhanced alternative splicing of FGFR2 to the mesenchymal isoform IIIc. BLM-induced lung fibrosis was also associated with the activation of TGF-β/Smad signaling. These findings have implications for rationally targetted strategies to therapeutically address IPF.

## Introduction

As its name suggests, idiopathic pulmonary fibrosis (IPF), a form of chronic, progressive, interstitial lung disease with a characteristic histopathological pattern of usual interstitial pneumonia [1], is a disease whose cause is poorly understood. IPF is the most common form of idiopathic interstitial pneumonia, with an estimated incidence of approximately 5–18 per 100000 [1–3]. The estimated prevalence of IPF is 1–63 per 100000 people, depending on the diagnostic criteria applied and the geographic location [1,4,5]. Lung transplantation is the only medical intervention known to extend life in patients with IPF, and the median survival of IPF patients is approximately 3–5 years [6]. Despite intense efforts to understand the pathogenesis of IPF, there is still an incomplete understanding of its risk factors, including the demographic, genetic, epigenetic, or environmental influences. Nevertheless, there is evidence that reactivation of developmental signaling pathways leads to alveolar epithelial cell damage, resulting in IPF [3,7,8]. Therefore, characterization of the molecular pathways and regulatory molecules involved in these pathways may enable the development of new therapies for IPF.

Exposure to bleomycin (BLM), a chemotherapeutic antibiotic, is associated with a high risk of developing lung fibrosis and has commonly been used in rodents to model pulmonary fibrosis for research purposes [9,10]. The inflammatory and fibrotic events in BLM-induced fibrosis are similar to those observed in human pulmonary fibrosis [11]. For example, intratracheal instillation of BLM into the lungs of mice causes the accumulation of pro-inflammatory cytokines, including interleukin-1, tumor necrosis factor-α, interleukin-6, and interferon-γ, followed by increased production of pro-fibrotic proteins such as transforming growth factor-β1 (TGF-β1), fibronectin, and procollagen-1 [9,10]. This model system has enabled significant discoveries related to the underlying causes of lung fibrosis, including the contribution of the epithelial to mesenchymal transition (EMT) of fibroblasts to myofibroblasts as well as the regulatory factors involved in EMT.

EMT plays a critical role in many pathophysiological conditions, including fibrosis and cancer progression [12]. Many regulatory and responsive factors of EMT have been identified, and a wealth of evidence suggests that targetting EMT is a promising strategy for disease management [12]. Accordingly, EMT has been proposed as a therapeutic target for the treatment of fibrotic and metastatic diseases [13,14]. TGF-β and its related signaling pathway are a major driver of EMT [13]. Disturbances in the orchestrated balance of epithelial factors also contribute to EMT, including disruption of the transcriptional and translational machinery, alterations in expression of noncoding RNAs, and changes in protein stability; one contributor in particular is the aberrant alternative splicing of fibroblast growth factor receptor 2 (FGFR2) mediated by epithelial splicing regulatory proteins (ESRPs) [15–17]. It has been shown that alternative splicing of FGFR2 plays an essential role in the regulation of cell proliferation, differentiation, migration, EMT, and embryonic development [15–18]. However, the role of the abovementioned factors in BLM-induced lung fibrosis has not been fully characterized.

Thus, the goal of the present study is to explore the underlying mechanisms of EMT-mediated lung fibrosis using BLM-induced lung fibrotic and transgenic mouse models, with a focus on the alternative splicing of FGFR2. Utilizing these models, we have shown that BLM regulates alternative splicing of FGFR2, leading to an increased conversion of epithelial isoform FGFR2 IIIb into mesenchymal isoform FGFR2 IIIc. This enhanced conversion of FGFR2 from epithelial to mesenchymal is associated with the activation of the TGF-β/Smad signaling pathway. Our findings suggest that TGF-β/Smad signaling contributes to the development of EMT by regulating FGFR2 IIIc alternative splicing in BLM-induced lung fibrosis.

## Materials and methods

### Cell culture

A549 cells were obtained from the American Type Culture Collection (ATCC, Manassas, VA, U.S.A.) and maintained in MEM/F12 medium supplemented with 1.5 mg/ml of sodium bicarbonate and 10% FBS (N-10) (Gibco, Carlsbad, CA, U.S.A.) in a 5% CO_2_ incubator at 37°C. Cells were seeded at a density of 0.7 × 10^5^ cells/well in six-well plates overnight. The culture medium was replaced with 2 ml fresh medium without serum for the treatment with BLM (20 µg/ml), bronchoalveolar lavage fluid (BALF, 200 µl), or TGF-β1 (5 ng/ml). The cells were collected accordingly and stored at −80°C for further analysis.

### Animal care

Kunming mice (male, 25 ± 2 g, 4–6 weeks), were used to establish the model of lung fibrosis and purchased from animal center of Daping Hospital (Chongqing, China). Kunming mice, a type of mouse commonly used in laboratory testing, first originated from Swiss mice. It is commonly used for toxicology and neurology studies owing to its high yield, good quality, resistance, and strong adaptability. Particularly, it is the most highly produced mice in China nowadays for laboratory research in pharmacology and toxicology studies [19,20]. The transgenic mice (male, 25 ± 2 g, 4–6 weeks) were used to study the EMT. All mice were housed in groups of four in plastic cages equipped with air filters, containing wood shaving and cotton bedding in standard animal facility (22–25°C and 12-h light/night cycle). All mice were allowed to acclimate at the facility for at least 1 week prior to experiment with free access to standard laboratory diet and water. The animal study protocol was approved by the Ethics Committee for Animal Experiments of the local and the Third Military Medical University (Chongqing, China). The animal experiments were performed in accordance with guidelines and regulations of Ethics Committee for Animal Experiments.

### Transgenic mice

The ROSA26 lacZ reporter (ROSA26R) mice (25 ± 2 g, 4–6 weeks) were purchased from the Model Animal Research Center of Nanjing University (Nanjing, China), and surfactant protein C (SPC)-Cre mice (25 ± 2 g, 4–6 weeks) were generously provided by Brigid L.M. Hogan at Duke University. The SPC-Cre and LacZ double transgenic mice (*n*=26) were bred via hybridization of ROSA26R mice and SPC-Cre mice. In brief, a loxP flanked transcriptional stop cassette was inserted downstream of a transcription start site at the ubiquitously expressed ROSA26 locus in ROSA26R mice such that the stop cassette blocks the expression of β-galactosidase (β-gal) from the downstream *LacZ* coding sequence in the intact condition. Upon exposure to Cre recombinase, the stop cassette is excised, leading to the recombination between the loxP sites and expression of the *LacZ* reporter. The double transgenic system provides an effective means of tracing cell lineage for the investigation of EMT. Transgenic mice were treated with 6 mg/kg BLM for 4 weeks, in 5- to 7-μm thick freshly frozen tissue sections, the LacZ expression was evaluated using colorigenic substrates for β-gal as previously described by Kim et al. [21]. The transgenic mouse study protocol was approved by the Ethics Committee for Animal Experiments of the local and the Third Military Medical University (Chongqing, China). The transgenic mouse experiments were performed in accordance with guidelines and regulations of Ethics Committee for Animal Experiments.

### Intratracheal instillation

Kunming mice (*n*=30) were used to establish the model of lung fibrosis via intratracheal instillation of BLM. In brief, mice were anesthetized with 0.1% pentobarbital and secured on an inclined plastic platform prior to BLM administration. Mice were dosed with 6 mg/kg of BLM or normal saline through the exposed trachea by a 1-cm incision on the ventral neck skin followed by suture repair of the incision. Mice were recovered and observed daily until their scheduled termination 1, 2, 3, or 4 weeks later. Six mice were killed at each end point. The BALF was collected via PBS (1 ml) perfusion. The final volume of 0.8 ml of BALF was collected and centrifuged at 4°C. The supernatant was collected and filtered via a 0.2-µm filter. The filtered BLAF was stored at −80°C for further experiment. Lungs were collected for further analysis.

### RNA isolation, reverse transcription, and qRT-PCR

Total RNA was extracted from collected lung tissue or A549 cells using the E.Z.N.A.™ Total RNA Kit II (OMEGA, CA, U.S.A.). The cDNA was prepared using the Superscript™ III First-Strand Synthesis system and Platinum PCR SuperMix (Invitrogen Carlsbad, CA, U.S.A.). qTR-PCR was performed using QUANTINOVA SYBR Green mix (QIAGEN, Germany) with ECO qRT-PCR detector (Gene Ltd.). Primer sequences are listed in Table 1.

**Table 1:** The sequence of primers

Gene	Sequence
Human *FGFR2*	Forward: 5′-TGGATCAAGCACGTGGAAAAGA-3′
	Reverse: 5′-GGCGATTAAGAAGACCCCTATGC-3′
Human *ESRP1*	Forward: 5′-CAGAGGCACAAACATCACAT-3′
	Reverse: 5′-AGAAACTGGGCTACCTCATTG G-3′
Human *ESRP2*	Forward: 5′-TGGTGTGGCCCTCTGTCTCAAC-3′
	Reverse: 5′-GCCCCCTGCAATCTTTACGAA-3′
Mouse *Fgfr2 IIIb*	Forward: 5′-CACTCGGGGATAAATAGCTCC-3′
	Reverse: 5′-AGATGACTGTCACCACCATGCA-3′
Mouse *Fgfr2 IIIc*	Forward: 5′-CGGTGTTAACACCACGGAC-3′
	Reverse: 5′-AGATGACTGTCACCACCATGCA-3′
Mouse *Esrp1*	Forward: 5′-GCCTGGCCTACAATACTGGAGTTA-3′
	Reverse: 5′-CTGGCCTGGTCATTTGTGTGT-3′
Mouse *Esrp2*	Forward: 5′-GGACTAGAAACAGATGCTACCGAAGA-3′
	Reverse: 5′-CTTCGAGAACAACTGACCATTGG-3′
Mouse *Gapdh*	Forward: 5′-GGTGAAGGTCGGTGTGAACG-3′
	Reverse: 5′-CTCGCTCCTGGAAGATGGTG-3′

### FGFR2 alternative splicing

The EGFR2 is approximately 365 bp in length. Enzyme AvaI can only digest the IIIb fragment, and cut two fragments of 118 and 249 bp in length, respectively; HicII can only excise the IIIc fragment and cut three fragments of 125, 119, and 120 bp in length, respectively [36]. The restriction fragments were separated on 1% agarose gel as described.

The alternative splicing of FGFR2 was examined in A549 cells. The cells were cultured in serum-free medium in the presence of TGF-β1 (5 ng/ml) or fibrotic lung tissue BALF (200 µl) for 48 h, then the total mRNA was collected for reverse transcript PCR. Primer sequences are listed in Table 1.

### Histopathologic study

The lung samples were collected for routine histopathologic examination. After flushing with cold PBS, the tissue was fixed in 4% paraformaldehyde for 72 h at 4°C. Four micrometer tissue sections were subjected to H&E and Masson’s staining, and followed by microscopic examination (Nikon Eclipse 50i; Kanagawa, Japan).

### Immunoassays

Immunohistochemistry and immunocytochemistry were performed to examine the expression of ESRP, E-cadherin, and vimentin in cells according to the manufacturer’s instructions. Western blotting assays were also performed using whole cell lysates. Cell lysates were prepared and separated by SDS/PAGE. Proteins were transferred on to PVDF membrane, and membranes probed primary antibodies including ESRP1/2 (anti-RBM35A+RBM35B, ab106555, Abcam), Smad (ab207447, Abcam), or p-Smad (sc-11769, Santa Cruz Biotechnology) overnight at 4°C, then blotted with corresponding secondary antibodies (ab136817 and ab6785, Abcam). Visualization was accomplished using BioRad ChemiDoc™ XRS system (Hercules, CA) with electrochemiluminescence substrate. Protein levels were normalized to match densitometric values of internal controls. In addition, ELISA was performed to examine the level of TGF-β1 (ab119557, Abcam) in BALF according to the manufacturer’s instructions.

### Statistics

Data are expressed as the mean ± S.D. Multiple comparisons were evaluated by one-way ANOVA followed by Tukey’s multiple comparisons. All assays were performed in triplicate. A value of *P*<0.05 was considered statistically significant.

## Results

### BLM induces lung fibrosis in mice

**Figure 1:**
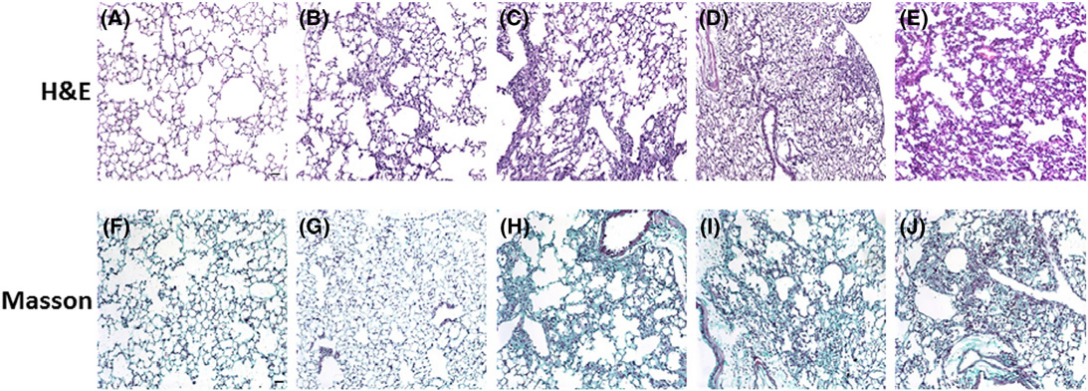
BLM induces lung fibrosis in mice Mice were treated with BLM at 6 mg/kg for 1, 2, 3, or 4 weeks. The tissues were processed and stained with H&E for the examination of tissue morphology and Masson’s trichrome for the identification of collagen. H&E staining: (**A**) control, (**B**) 1-week, (**C**) 2-week, (**D**) 3-week, (**E**) 4-week. Masson’s staining: (**F**) control, (**G**) 1-week, (**H**) 2-week, (**I**) 3-week, (**J**) 4-week. Scale bar: 100 µm.

First, BLM was employed to establish the lung fibrosis model in mice. Kunming mice were administered BLM at a dosage of 6 mg/kg over 4 weeks. Lung tissues were collected after 1, 2, 3, or 4 weeks of administration of BLM. Histological changes which were consistent with fibrosis were observed. Compared with the control group, BLM treatment induced marked distortion of lung architecture, including development of irregular alveolar septa after 3–4 weeks, which is consistent with alveolar damage (Figure 1A–E). Additionally, BLM treatment resulted in increased collagen deposition in a time-dependent manner, as showed by Masson’s staining, which indicates the occurrence of fibrosis (Figure 1F–J).

### BLM promotes EMT during the lung fibrosis process in mice

**Figure 2:**
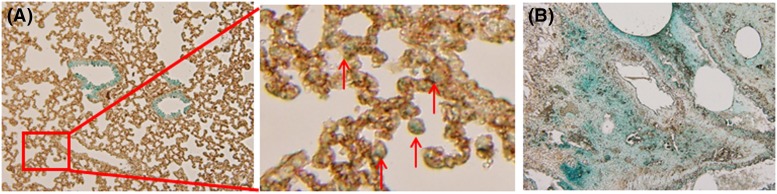
BLM promotes EMT during lung fibrosis in SPC-Cre and LacZ double transgenic mice SPC-Cre and LacZ double transgenic mice were treated with BLM at 6 mg/kg for 4 weeks and the expression of LacZ was evaluated via colorigenic substrates for β-gal. (**A**) Control, the red arrow indicates alveolar epithelial type II cells, (**B**) BLM 4-week. Scale bar: 500 µm.

Following the establishment of the BLM-induced lung fibrosis model, we aimed to measure EMT during the course of lung fibrosis. To study the transition of alveolar bronchial epithelial cells in BLM-exposed mice, we subsequently employed SPC-Cre and LacZ double transgenic mice to facilitate cell lineage tracing. These mice stably express SPC and LacZ in alveolar epithelial cells (Figure 2), allowing for the identification of all daughter cells by β-gal staining. Transgenic mice were treated with 6 mg/kg BLM for 4 weeks, and the histologic distribution of LacZ expression was evaluated using colorigenic substrates for β-gal. In untreated mice, LacZ localized to alveolar bronchial epithelial cells and alveolar epithelial type II cells (Figure 2A), whereas in BLM-treated mice LacZ was translocated to alveolar mesenchymal cells (Figure 2B). Taken together, these results are consistent with the mobilization of alveolar bronchial epithelial cells into the lung parenchyma by EMT.

### BLM decreases the level of ESRP1 and alters the location of ESRP in fibrotic lung tissue

**Figure 3:**
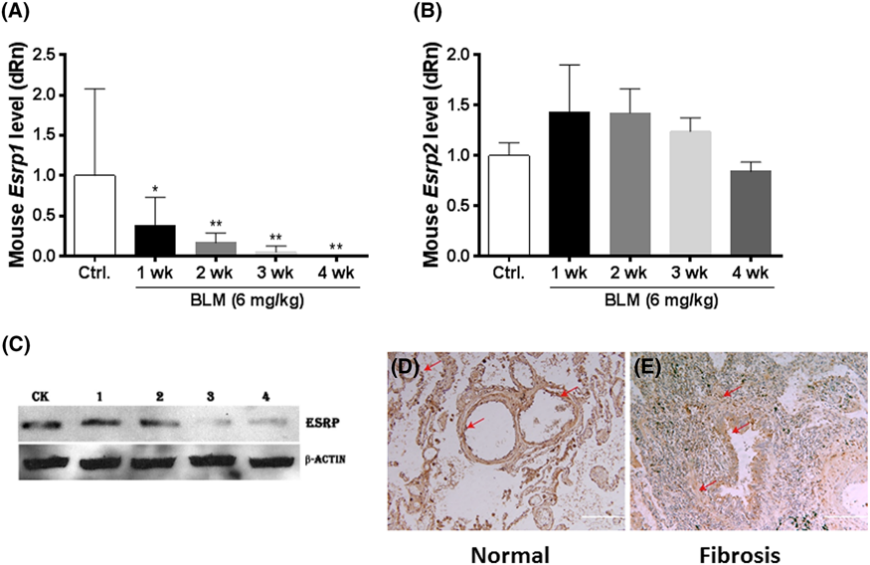
BLM decreases the level of ESRP1 and alters the location of ESRP in fibrotic lung tissue (**A**,**B**) Fibrotic lung tissues were collected and the mRNA levels of *Esrp1* (A) and *Esrp2* (B) were evaluated. (**C**) The protein level of ESRP was detected by Western blotting, with β-actin serving as an internal control. Mice were treated with BLM for 1, 2, 3, or 4 weeks and then compared with the untreated group (CK). (**D**,**E**) Expression and localization of ESRPs in normal or fibrotic human lung tissues were examined. The red arrow indicates alveolar and bronchial epithelial cells expressing ESRP. Scale bar: 500 µm. *, *P*<0.05; **, *P*<0.01. The data were obtained from three independent assays and analyzed using one-way ANOVA followed by Tukey’s multiple comparison.

After we observed EMT during the course of lung fibrosis, we explored the possible mechanisms of EMT with a focus on alternative splicing. ESRPs are cell type-specific regulators of mRNA splicing for transcripts important for EMT, such as FGFR2, CD44, p120-Catenin (CTNND1), and hMena (ENAH) [16,22]. We hypothesized that ESRPs may regulate aspects of EMT in our lung fibrosis models. Therefore, we first evaluated the level of *Esrp1/2*. In comparison with the control, BLM treatment significantly reduced the level of *Esrp1* in fibrotic lung tissues in a time-dependent manner, as observed by qRT-PCR (Figure 3A), but there was no significant change in the level of *Esrp2* (Figure 3B). Analysis of the protein level for ESRP1/2 showed that BLM markedly decreased the level of ESRP1/2 in fibrotic lung tissues compared with control (Figure 3C).

To determine if these findings also apply to human lung tissue, the expression and localization of ESRP1/2 were examined in fibrotic lung tissue specimens. As shown in Figure 3D, in normal human lung tissue, ESRP1 and 2 were localized to alveolar and bronchial epithelial cells; however, in fibrotic tissues, irregular alveoli and hyperplastic interstitium were observed. Furthermore, there was decreased expression of ESRP1/2 in bronchial epithelial cells and at the surface of the compressed alveolar cells (Figure 3E).

**Figure 4:**
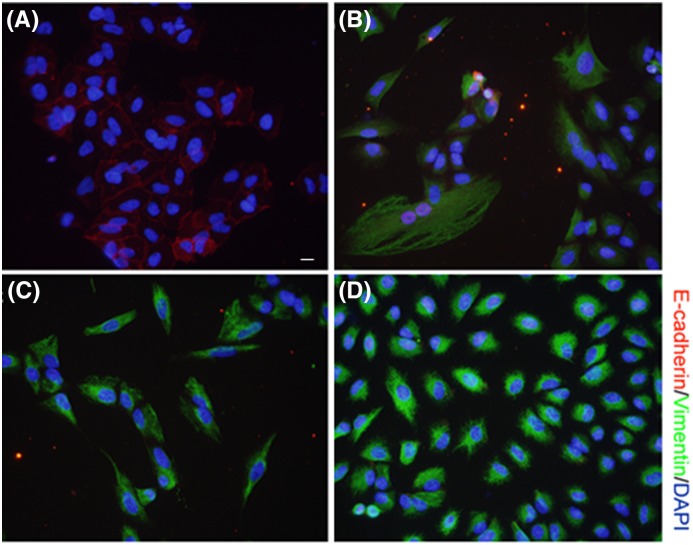
BLM induces EMT in A549 cells Cells were exposed to (**A**) vehicle, (**B**) BLM (20 µg/ml), (**C**) fibrotic lung tissue BALF (200 µl), or (**D**) TGF-β1 (5 ng/ml) for 24 h and the expression level of E-cadherin (red signal) and vimentin (green) was examined by immunofluorescence. Scale bar: 5 µm.

**Figure 5:**
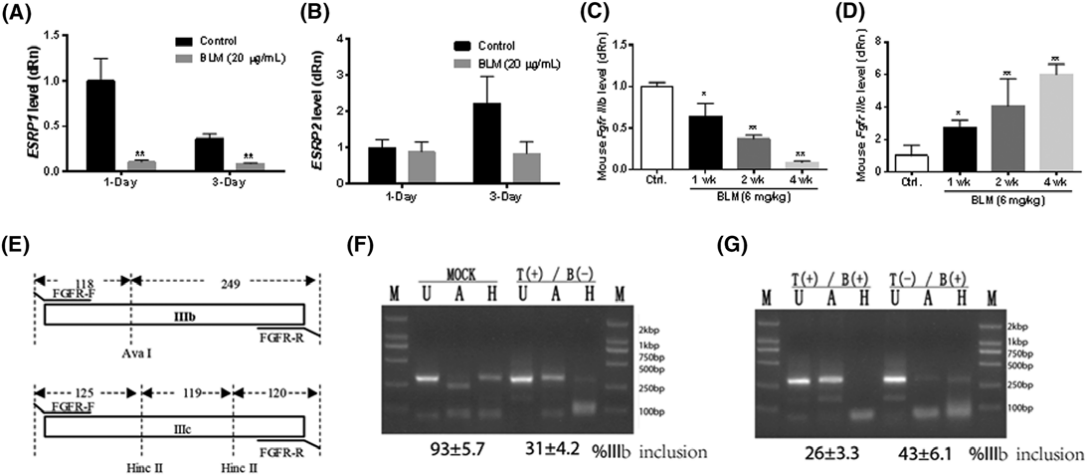
BLM reduces the level of ESRP1 in A549 cells and BLM enhances the alternative splicing of FGFR2 IIIc in fibrotic lung tissue mRNA levels of *ESRP1* (**A**) and *ESRP2* (**B**) were evaluated by qRT-PCR. (**C**,**D**) Effect of BLM on Fgfr2 splicing. The levels of Fgfr2 IIIb (C) and IIIc (D) were evaluated. (**E**) Experimental strategy used to differentiate the epithelial isoform FGFR2 IIIb from the mesenchymal isoform FGFR2 IIIc. (**F**,**G**) Effects of BLM on the alternative splicing of FGFR2 during BLM-induced EMT in A549 cells. T, TGF-β1; B, BALF; U, untreated; A, AvaI; H, HincII, M, DL2000. *, *P*<0.05; **, *P*<0.01. Data are from three independent assays and analyzed by one-way ANOVA followed by Tukey’s multiple comparison.

Finally, the effects of fibrogenic stimuli were studied in A549 cells, a commonly used *in vitro* model of Type II alveolar epithelium [23], to evaluate the occurrence of EMT and to study the extent of ESRP1 involvement in EMT. A549 cells were exposed to BLM (20 µg/ml), fibrotic lung tissue BALF (200 µl), or TGF-β1 (5 ng/ml) for 24 h. All three stimuli significantly induced EMT in A549 cells, as evidenced by the dramatic decrease in the expression of E-cadherin (red signal) and increase in the expression of vimentin (green signal) (Figure 4A–D). Furthermore, BLM (20 µg/ml) significantly decreased the level of ESRP1 in A549 cells after 1 or 3 days of treatment (Figure 5A), but there was no statistically significant change in the level of ESRP2 (Figure 5B). These results are in agreement with the observations in mice. Altogether, these data suggest that the expression of ESRP1 is correlated with EMT *in vivo* and *in vitro*.

### BLM enhances alternative splicing of mesenchymal isoform FGFR2 IIIc

To further understand the role of ESRP1 in EMT and lung fibrosis, alterative splicing of FGFR2 was examined. ESRP1 controls splicing during EMT by regulating the alternative splicing of FGFR2, which results in two cell type-specific isoforms: IIIb (epithelial) and IIIc (mesenchymal) [22]. To evaluate the role of ESRP1 and the effects of BLM on Fgfr2 IIIb and IIIc splicing in fibrotic lung tissues, we measured the mRNA level of Fgfr2 isoforms. In comparison with the control group, BLM treatment significantly decreased the mRNA level of the epithelial isoform *Fgfr* IIIb (Figure 5C), while increasing the mRNA level of the mesenchymal isoform *Fgfr* IIIc (Figure 5D). To further evaluate the generalizability of the effects of BLM on alternative splicing of FGFR2 during BLM-induced EMT, we examined the splicing of FGFR2 in A549 cells (Figure 5E). As shown in Figure 5F, qRT-PCR showed that TGF-β1 induced a transition from FGFR2 IIIb to IIIc in A549 cells. A similar phenomenon was observed in fibrotic lung tissue BALF (Figure 5G). Coincubation of TGF-β1 and fibrotic lung tissue BALF further enhanced the FGFR2 IIIc isoform switching effect, compared with TGF-β1 or fibrotic lung tissue BALF alone (Figure 5G). Taken together, these results suggest that BLM, with input from TGF-β, induces FGFR2 IIIb to IIIc isoform switching during EMT in fibrotic lung tissues.

### BLM promotes TGF-β/Smad signaling pathway

**Figure 6:**
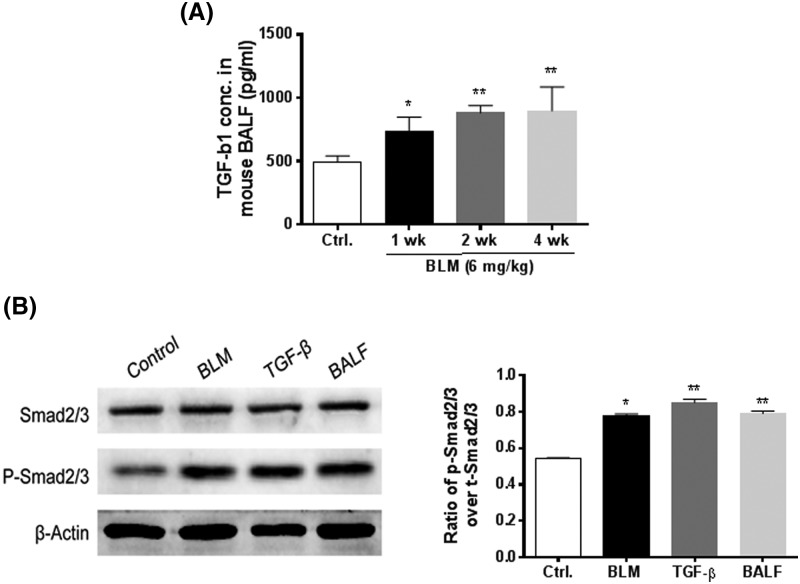
BLM promotes TGF-β/Smad signaling (**A**) TGF-β1 levels were examined in BALF using ELISA. (**B**) Expression and phosphorylation of Smad2/3 were examined by Western blotting. *, *P*<0.05; **, *P*<0.01. The data were obtained from three independent assays and analyzed by one-way ANOVA followed by Tukey’s multiple comparison.

To further evaluate the contribution of TGF-β to BLM-induced EMT during lung fibrosis, signaling analysis was performed in A549 cells after fibrinogenic stimulation. First, the level of TGF-β1 was determined in BALF using ELISA. As shown in Figure 6A, BLM treatment significantly increased the level of TGF-β1 in BALF compared with that in the control. On the other hand, exposure of cells to BLM (20 µg/ml), fibrotic lung tissue BALF (200 µl), or TGF-β1 (5 ng/ml) for 24 h activated TGF-β/Smad signaling, as demonstrated by the increase in phosphorylation of Smad2/3 (Figure 6A). Notably, BLM exhibited a similar effect to TGF-β1 on the activation of TGF-β/Smad signaling. Taken together, these results demonstrate that TGF-β/Smad signaling is involved in BLM-induced EMT in lung fibrosis.

## Discussion

A wealth of evidence shows that IPF has become an important public health problem in terms of increasing incidence, poor prognosis, and unresponsiveness to traditional therapies [1,3,7]. Although considerable efforts have been devoted to understanding the factors involved in the etiology and pathogenesis of IPF, such as occupational and environmental exposure, tobacco consumption, gastroesophageal reflux, genetic factors, and pathologically activated molecular pathways, the understanding of the pathogenesis of IPF is still incomplete. Therefore, more efforts are needed to delineate the specific clinical and pathologic characteristics of IPF to better understand the onset, development, and progression of IPF, particularly to facilitate the development of novel and efficacious therapies for IPF.

In the present study, in order to explore the possible mechanisms of lung fibrosis and for the benefit of further translational studies on IPF, we employed different mice models to study EMT in lung fibrosis. Utilizing the SPC-Cre transgenic mouse model, we have shown that BLM induces EMT during lung fibrosis by promoting the transition of epithelial cells into alveolar mesenchymal or mesenchymal-like states. Mechanistically, this occurs through BLM-mediated negative regulation of ESRP1 expression, leading to changes in the alternative splicing of FGFR2 in favor of the mesenchymal isoform FGFR2 IIIc, as opposed to the epithelial isoform of FGFR2 IIIb. In addition, the effects of BLM on lung fibrosis are associated with changes in the activity of TGF-β/Smad signaling.

EMT exerts a pivotal role in many pathophysiological processes including fibrosis [24], with its prime features being the loss of the epithelial phenotype and gain of the mesenchymal phenotype. For example, epithelial cells lose their polarization and specialized junction structures, undergo cytoskeletal reorganization, and acquire the morphological and functional features of mesenchymal-like cells [25,26]. In the present study, the SPC-Cre and LacZ double transgenic mouse model has clearly demonstrated that the alveolar mesenchymal cells derived from alveolar bronchial epithelial cells, as evidenced by β-gal expression in EMT cells. This double transgenic animal model provides an effective approach to tracing the origin of mesenchymal or mesenchymal-like cells. It suggests that the epithelial cells transition to mesenchymal or mesenchymal-like cells in BLM-induced lung fibrosis.

Mechanistically, ESRPs (ESRP1 and ESRP2) are cell type-specific regulators of transcription that switch splicing during EMT [16,22,27]. ESRPs are essential regulators of FGFR2 splicing that results in the two isoforms FGFR2 IIIb and IIIc [22]. FGFR2 exhibits a cell type-specific expression profile, by which the tight regulation of expression of the mutually exclusive exons IIIb or IIIc results in either epithelial or mesenchymal isoform, respectively [22]; it has been reported that TGF-β-induced EMT involves isoform switching of FGFR2 via alternative splicing [28]. In the present study, our findings have shown an increased level of TGF-β during BLM-induced lung fibrosis and BLM-induced alternative splicing of FGFR2. Intriguingly, BLM-induced isoform switching of FGFR2 is similar to that seen in TGF-β-treated cells. Moreover, BLM dramatically activates the TGF-β/Smad signaling pathway that underlies the mechanism for BLM-induced EMT during lung fibrosis. More interestingly, given the critical role of ESRPs in EMT [16,22,29,30], they may be potential therapeutic targets in the treatment of fibrosis and cancer. Indeed, recent clinical and preclinical data have shown a tumor-suppressive effect of ESRP2 in clear cell renal cell carcinoma [31]. On the other hand, although there are data to support the critical role of ESRPs in cancer in preclinical and clinical settings, it will be challenging to characterize ESRPs as drug targets due to the different mechanisms of ESRP1 and ESPR2 in the regulation of EMT and the lack of a protein crystal structure [32]. Thus, it is inevitable that the difference in the regulatory mechanism will need to be addressed via the analysis of determinants at the protein structural level to pave the way for drug development.

Additional studies will be required to clarify the applicability of the abovementioned results to human IPF. Although BLM-induced lung injury in mice is the most commonly used model to mimic pulmonary fibrosis and study the molecular pathways that are involved in humans [33,34], some studies have shown that the clinical and pathological changes in BLM models can differ from IPF in humans. While many of the histological hallmarks that are seen in BLM-treated animals, including intra-alveolar buds, mural incorporation of collagen, and obliteration of the alveolar space, are similar to those observed in IPF patients [33,35], the location of fibrosis can differ [36,37]. Our findings have shown that the intratracheal instillation of BLM significantly distorted the organization of alveolar structures and induced the accumulation of collagen in lung tissues, mimicking the development of fibrosis in human lungs. Nevertheless, caution should be employed when attempting to extrapolate observations in animals to those in human fibrotic lung diseases.

In conclusion, our results reveal that BLM-induced lung fibrosis involves EMT. BLM suppresses the expression of ESRP1, leading to the induction of isoform switching of FGFR2 via alternative splicing. The activation of the TGF-β/Smad signaling pathway provides an explanation for the effects of BLM in the process of lung fibrosis.

## Abbreviations

β-gal: β-galactosidase
BALF: bronchoalveolar lavage fluid
BLM: bleomycin
CTNND1: Catenin Delta 1
EMT: epithelial to mesenchymal transition
ESRP: epithelial splicing regulatory protein
FGFR2: fibroblast growth factor receptor 2
IPF: idiopathic pulmonary fibrosis
ROSA26R: ROSA26 lacZ reporter
SPC: surfactant protein C
TGF-β1: transforming growth factor-β1
